# S phase block following *MEC1^ATR^* inactivation occurs without severe dNTP depletion

**DOI:** 10.1242/bio.015347

**Published:** 2015-11-24

**Authors:** Caroline Earp, Samuel Rowbotham, Gábor Merényi, Andrei Chabes, Rita S. Cha

**Affiliations:** 1 Stem Cell Biology and Developmental Genetics, National Institute for Medical Research, MRC, London NW7 1AA, UK; 2 Department of Medical Biochemistry and Biophysics, Umeå University, Umeå SE 901 87, Sweden; 3 North West Cancer Research Institute, School of Medical Sciences, Bangor University, Bangor LL57 2UW, UK

**Keywords:** Mec1, ATR, Sml1, RNR, dNTP, DNA replication, Replication arrest

## Abstract

Inactivation of Mec1, the budding yeast ATR, results in a permanent S phase arrest followed by chromosome breakage and cell death during G2/M. The S phase arrest is proposed to stem from a defect in Mec1-mediated degradation of Sml1, a conserved inhibitor of ribonucleotide reductase (RNR), causing a severe depletion in cellular dNTP pools. Here, the casual link between the S phase arrest, Sml1, and dNTP-levels is examined using a temperature sensitive *mec1* mutant. In addition to S phase arrest, thermal inactivation of Mec1 leads to constitutively high levels of Sml1 and an S phase arrest. Expression of a novel suppressor, *GIS2,* a conserved mRNA binding zinc finger protein, rescues the arrest without down-regulating Sml1 levels. The dNTP pool in *mec1* is reduced by ∼17% and *GIS2* expression restores it, but only partially, to ∼93% of a control. We infer that the permanent S phase block following Mec1 inactivation can be uncoupled from its role in Sml1 down-regulation. Furthermore, unexpectedly modest effects of *mec1* and *GIS2* on dNTP levels suggest that the S phase arrest is unlikely to result from a severe depletion of dNTP pool as assumed, but a heightened sensitivity to small changes in its availability.

## Abstract (precis)

**Summary:** This study, using a temperature sensitive *mec1* mutant, reveals that inactivation of Mec1 leads to S phase arrest, and that genome duplication in the absence of Mec1/ATR is exquisitely sensitive to small changes in dNTP levels.

## INTRODUCTION

Budding yeast Mec1 belongs to the conserved ATM/ATR family of signal transducers involved in a range of processes, including DNA damage repair, checkpoint response, cell cycle regulation, and meiosis (Kato and Ogawa, 1994; Weinert et al., 1994; Abraham, 2001; Carballo et al., 2008). In addition, Mec1 and its mammalian counterpart ATR, are essential during unperturbed proliferation, whereby their inactivation leads to permanent DNA replication block followed by a fatal mitotic catastrophe in the respective organism (Brown and Baltimore, 2000; Casper et al., 2002; Cha and Kleckner, 2002; Eykelenboom et al., 2013).

The replication block in *mec1* cells was proposed to stem from a defect in the Mec1-Rad53-Dun1 dependent removal of Sml1 at the onset of S phase (Zhao et al., 1998, 2001; Zhao and Rothstein, 2002). Sml1 is an inhibitor of the ribonucleotide reductase (RNR), which catalyses the rate limiting step in dNTP synthesis (Desany et al., 1998; Zhao et al., 1998, 2001; Zhao and Rothstein, 2002). Rad53, a homolog of mammalian CHEK2, is an essential downstream effector kinase of Mec1 (Allen et al., 1994; Matsuoka et al., 1998). Dun1 is another serine/threonine kinase and responsible for Sml1 phosphorylation and degradation (Zhao et al., 2001; Zhao and Rothstein, 2002). According to this view, the Mec1-Rad53-Dun1-dependent Sml1 removal and ensuing RNR activation would promote the dNTP production. In support for this view, it was shown that dNTP levels in *mec1-* or *rad53-*hypomorphs and a *dun1*Δ strain were reduced by as much as 46% compared to a *MEC1* control strain (Zhao et al., 2001; Fasullo et al., 2010; Hoch et al., 2013).

Notably, however, nearly all analyses on a lethal *mec1* allele [e.g. *mec1*Δ or *mec1-kd* (kinase dead)] have been performed in a strain background that was either deleted for *SML1* or over-expressing *RNR1*, a requirement for maintaining viability of a mutant lacking Mec1's essential function (e.g. Desany et al., 1998; Zhao et al., 1998). As a result, while it is clear that absence of Mec1 causes dNTP pool to decrease, the true extent of the reduction and whether it would be sufficient to account for the replication arrest remain elusive. Here, we addressed these questions utilizing a temperature sensitive mutant, *mec1-4,* which maintains its viability at permissive temperature in an otherwise wild-type background, circumventing the need to exogenously manipulate Sml1 and/or RNR activity (Cha and Kleckner, 2002).

## RESULTS AND DISCUSSION

**Fig. 1.**
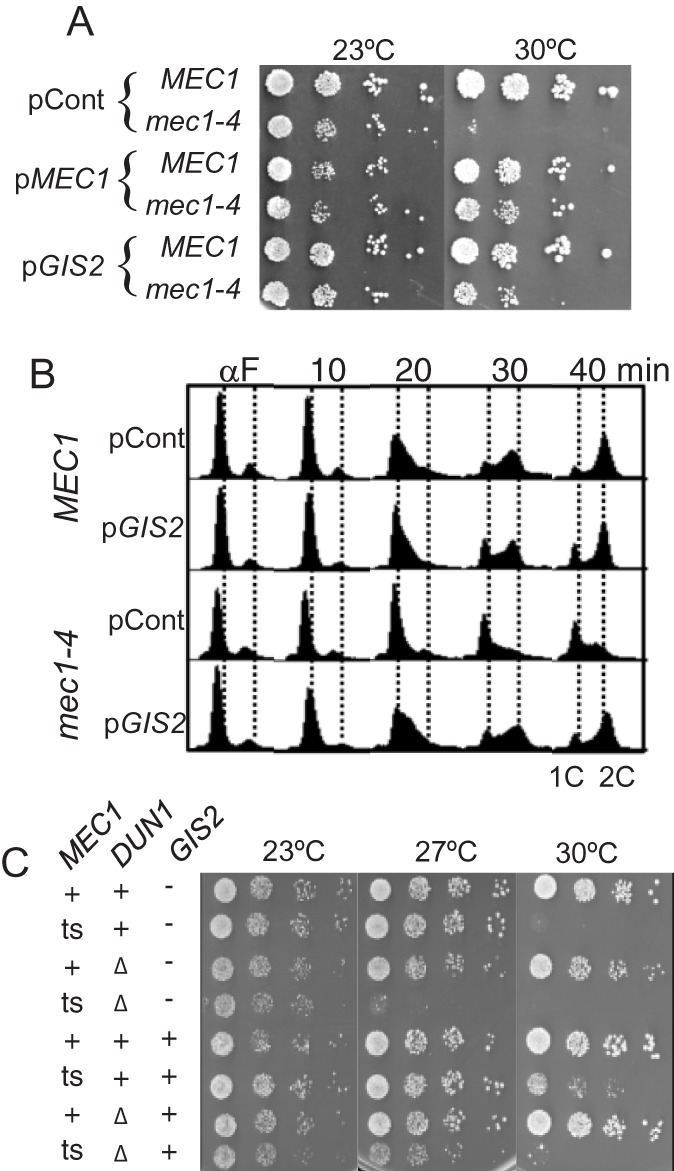
***GIS2* suppression of *mec1* lethality and replication defect.** (A) *MEC1* or *mec1-4* strains carrying the indicated plasmids were grown at permissive temperature (23°C) to mid-log phase before being diluted to OD600 of 0.5. Ten-fold serial dilutions were spotted and incubated at the indicated temperature for two days. pCont, YEp24 plasmid; p*MEC1* or p*GIS2*, YEp24 carrying either *MEC1* or *GIS2,* respectively. (B) Log phase cultures of strains with the indicated genotypes were α-factor arrested at permissive temperature (23°C) and released into fresh YPD at 30°C. Samples were collected every 10 min and subjected to FACS analysis. The positions of 1- or 2-cell DNA content (1C or 2C) are as indicated. (C) Strains with the indicated genotypes were subjected to spot-test as described in A. ‘+’ or ‘*ts*’ in the *MEC1* column corresponds to *MEC1* or *mec1-4* allele, respectively. ‘+’ or ‘Δ’ in the *DUN1* column corresponds to *DUN1* or *dun1*Δ allele, respectively. ‘+’ or ‘−’ in the *GIS2* column corresponds to p*GIS2* or pCont, respectively.

We began the analysis by performing a multi-copy suppressor screen for *mec1-4* (Fig. S1). The screen identified *GIS2* (glucose inhibition of gluconeogenic growth suppressor 2) as a novel suppressor (Fig. 1A): The only other suppressors identified were *MEC1* and *RNR1* (Fig. S1). *GIS2* was originally isolated based its role in alternative carbon source utilization (Balciunas and Ronne, 1999). Subsequently, it was shown to encode a conserved zinc finger protein, whose orthologs include the fission yeast Byr3, identified as a negative regulator of the RAS/PKA pathway (Wang et al., 1991) and CNBP/ZNF9, an essential mammalian protein, implicated in myotonic dystrophy type 2 (Rajavashisth et al., 1989; Liquori et al., 2001).

To rule out the possibility that *GIS2* was an allele specific suppressor*,* we examined its effects on a different *mec1* allele, *mec1-40*: While *mec1-4* contains a single amino acid alteration in the conserved kinase domain, *mec1-40* carries an alteration in the N-terminal HEAT (Huntington, elongation factor 3, protein phosphatase 2A, Tor1) repeat domain (Perry and Kleckner, 2003; E. Waskiewicz and R.C., unpublished results). Introduction of a multi-copy plasmid carrying *GIS2* (p*GIS2*) also suppressed *mec1-40* temperature sensitivity*,* demonstrating that the suppression was not allele-specific (Fig. S2A); however, it was not able to rescue a null (*mec1*Δ) or a kinase dead (*mec1-kd*) (data not shown). Notably, *GIS2* did not rescue lethality conferred by temperature sensitive alleles of *YCG1*, *TOP2*, *ESP1* or *DBF4*, encoding for a condensin subunit, topoisomerase II, separase, or the regulatory subunit of Cdk7-Dbf4 kinase, respectively; thus, *GIS2* is not a suppressor of general temperature sensitivity (Fig. S2B).

To test whether the *GIS2* suppression was mediated by restoring Mec1's function in responding to replication stress or DNA damage, we assessed the effects of p*GIS2* on sensitivity of *mec1-4* to hydroxyurea (HU) or methyl methanesulfonate (MMS), respectively. *GIS2* did not rescue the drug sensitivity (Fig. S2C), suggesting that the suppression was independent of the role of Mec1 in mediating responses to HU or MMS.

The effects of *GIS2* on S phase progression were assessed. In a *MEC1* strain carrying either p*GIS2* or a control YEp24 plasmid (pCont), genome duplication was initiated and completed within 40 min following α-factor arrest/release (Fig. 1B). A *mec1-4* strain carrying pCont initiated genome duplication but failed to complete, in agreement with previous reports (Cha and Kleckner, 2002; Hashash et al., 2012). In contrast, DNA replication in the same *mec1-4* strain carrying p*GIS2* was completed by t=40 min. We infer that the *GIS2* rescue of *mec1* lethality is mediated by promoting efficient genome duplication, thereby averting the downstream fatal mitotic catastrophe.

To test whether the *GIS2* suppression was dependent on the Mec1-Rad53-Dun1 pathway (Zhao et al., 2001; Zhao and Rothstein, 2002), we assessed the effects of *GIS2* on a *mec1-4 dun1*Δ double mutant (Fig. 1C). Deletion of *DUN1* shows synthetic growth defects with hypomorphic *mec1* mutants (e.g. Zhao and Rothstein, 2002). Similarly, we observed synthetic interaction between *DUN1* and *mec1-4,* whereby *dun1*Δ lowered restrictive temperature of a *mec1-4* strain from 30°C to 27°C (Fig. 1C). Nevertheless, p*GIS2* improved viability of a *mec1-4 dun1*Δ mutant at 27°C, indicating that the suppression did not require the Mec1-Rad53-Dun1 signalling.

**Fig. 2.**
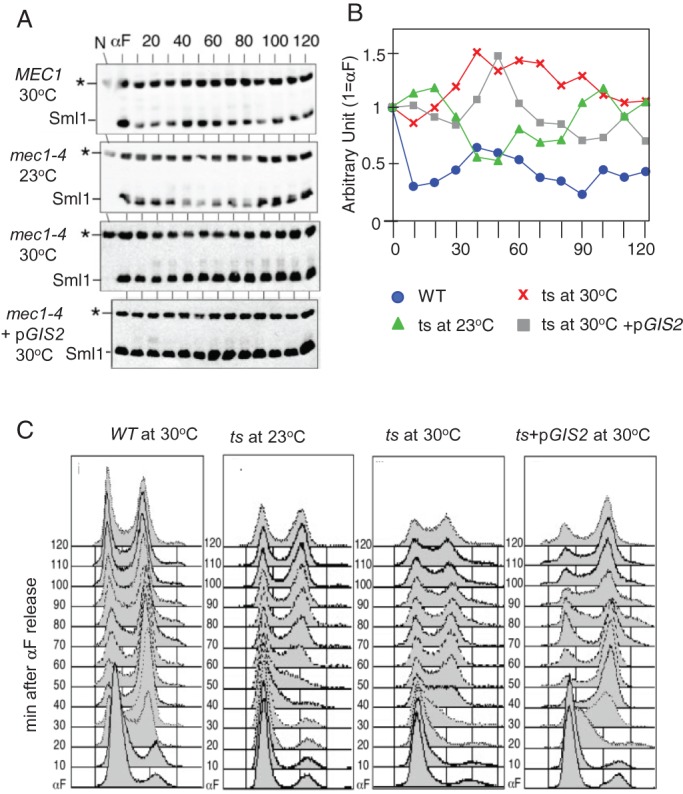
**Effects of *GIS2* on S phase progression and cell-cycle-dependent fluctuation in Sml1 levels.** (A) Strains with the indicated genotypes were α-factor arrested at 23°C and released into fresh YPD at either 23°C or 30°C. Western blot analysis using an α-MYC antibody was performed to detect MYC-Sml1. * indicates non-specific band used as a loading control; N, no-tag control sample. (B) The amounts of 3MYC-Sml1 in the western blots were quantified and normalised to the non-specific band (*) in the corresponding lane. These values were normalised to the value at t=0 (‘αF’) in each culture as a means to assess cell cycle dependent fluctuation in Sml1 levels. (C) Results of FACS analysis on samples analysed in A, *WT* corresponds to *MEC1*, *ts* corresponds to *mec1-4*, *ts*+p*GIS2* corresponds to *mec1-4*+p*GIS2*.

Next, we assessed the effects of p*GIS2* on steady-state Sml1 levels. During unchallenged proliferation, Sml1 undergoes S-phase- and *MEC1/RAD53/DUN1-*dependent downregulation (e.g. Zhao et al., 2001). As expected, we observed a notable reduction in the Sml1 levels in a *MEC1* strain between t=10-30 and 70-90 min following an α-factor arrest/release, corresponding to the first and presumably the second round of S phase, respectively (Fig. 2; Fig. S3). A similar S-phase-dependent reduction in Sml1 levels was observed in a *mec1-4* culture released at 23°C between t=40 and 80 min (Fig. 2). The notable delay in the timing is likely due to the lower temperature utilized to maintain viability of the mutant. The latter confirms that *mec1-4* cells are proficient in promoting the S-phase-dependent Sml1 destruction at permissive temperature. At 30°C, however, Sml1 levels in the mutant continued to increase and were maintained at high levels despite the fact that the cells were in S phase (Fig. 2; Fig. S3). Introduction of p*GIS2* promoted efficient genome duplication in the *mec1-4* strain at 30°C (Fig. 2C). Remarkably, however, the Sml1 levels in the latter did not decrease, but increased, during genome duplication (Fig. 2). The current observation eliminates Sml1 downregulation as a mechanism underlying the *GIS2* suppression. Furthermore, it demonstrates that the replication defect following Mec1 inactivation can be decoupled from Sml1 stabilization.

**Fig. 3.**
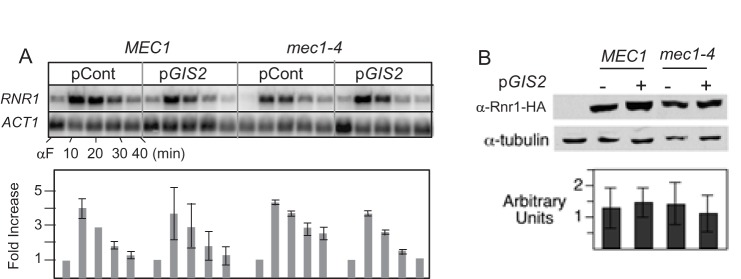
**Effects of *GIS2* on levels of the *RNR1* transcripts and proteins.** (A) Effects of *GIS2* on G1/S transition-dependent *RNR1* induction. *MEC1* or *mec1-4* strains carrying either pCont or p*GIS2* were released from α-factor arrest at 23°C into fresh YPD at 30°C. Samples were collected and subjected to FACS (Fig. 1B) and northern blot analysis (upper panel) using *RNR1* as probe. *ACT1*, encoding actin, is used as a loading control. Lower panel: the *RNR1* signals were quantified and normalized to the *ACT1* signal in corresponding sample. For each strain, the normalized values were then expressed relative to the values obtained for the α-factor sample, which was set to 1. The average of two independent experiments is shown. The error bars show ±s.e.m. (B) Effects of *GIS2* on steady state Rnr1-HA levels. *MEC1* or *mec1-4* strains carrying either pCont or p*GIS2* were grown to mid-log phase at 23°C before being shifted to 30°C for four hours. Upper panel: western blot analysis was performed using an α-HA and an α-tubulin antibodies. Lower panel: average levels of the HA-Rnr1 protein, normalized to the tubulin loading control, and calculated from four independent experiments. The error bars show ±s.e.m.

To test whether the *GIS2* suppression might be mediated by an increase in RNR activity, we assessed its effects on *RNR1* transcription induction at the onset of S phase (Fig. 3A). In all strains, the level of *RNR1* transcripts following α-factor arrest/release peaked at the first time point, t=10 min. The levels in the WT and *mec1-4*+p*GIS2* strains gradually decreased back to the basal level by 40 min (Fig. 3A), coinciding with the completion of bulk genome duplication in these cultures (Fig. 1B). Importantly however, p*GIS2* did not increase the level or duration of *RNR1* mRNA induction the *mec1-4* culture (Fig. 3A). We also assessed effects of p*GIS2* on levels of Rnr1 protein as well as *RNR2*, *3*, and *4* transcripts, where no noticeable difference was observed (Fig. 3B; Fig. S3). Taken together, we conclude that the *GIS2* suppression is not mediated by an increase in RNR expression.

**Fig. 4.**
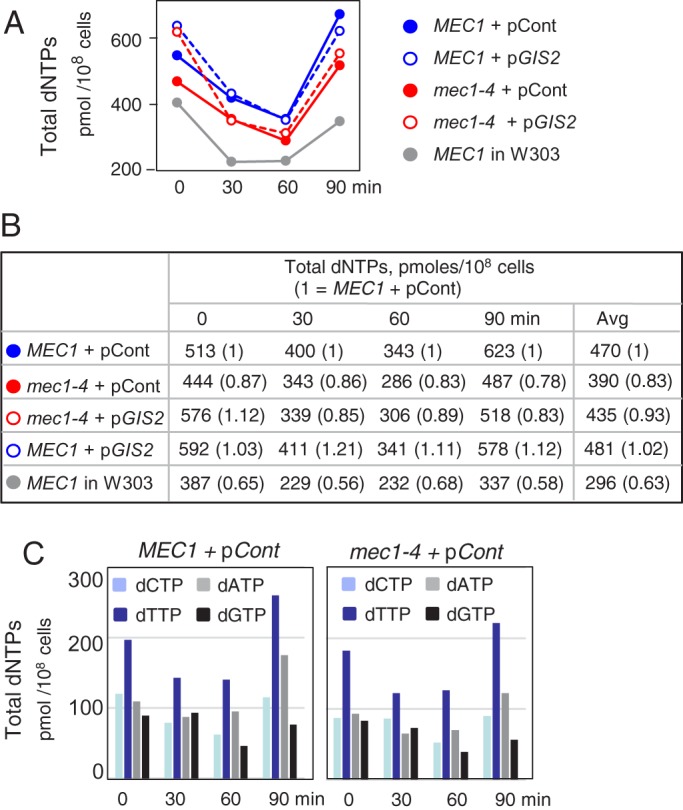
**Steady state dNTP levels in *MEC1* and *mec1-4* strains carrying pCont or p*GIS2*.** (A,B) Strains of the indicated genotypes were grown to mid-log phase at 23°C before being shifted to 30°C. Samples were collected at the specified times and analysed for levels of dATP, dCTP, dGTP, and dTTP. Shown in B are the total dNTP concentrations in each sample. For concentrations of each dNTP, see panel C and Fig. S5. All strains are in a SK1 background except for the ‘Control’, which is a *MEC1* strain in a W303 background. (C) Concentration of each of the four dNTPs in *MEC1* and *mec1-4* following a shift to 30°C.

In yeast, additional mechanisms of controlling dNTP production exist; for example, the dATP feedback inhibition of RNR and regulation of Rnr1, 2, 3, and 4 sub-cellular localization (Chabes et al., 2003; Yao et al., 2003; Lee et al., 2008). Instead of testing potential involvement of each of these mechanisms, we decided to directly assess the effects of *GIS2* on dNTP pools. Following a temperature shift from 23°C to 30°C, both *MEC1* and *mec1-4* cultures exhibited a transient reduction in the dNTP levels followed by a recovery (Fig. 4A). The dNTP levels in a *mec1-4* strain transformed with pCont were reduced to ∼83% of a *MEC1* control strain (Fig. 4B). Ectopic expression of *GIS2* led to an increase; however, the extent was limited, restoring dNTP levels to only ∼93% of the control. Thus, the replication arrest and its rescue conferred by Mec1 inactivation and *GIS2,* respectively, are both accompanied by unexpectedly modest changes in dNTP levels. The current observations are reminiscent of a *rad53* allele lacking key Mec1 phosphorylation sites, which was similarly shown to reduce dNTP pool by only ∼15% (Hoch et al., 2013).

dNTP levels in *mec1-* or *rad53-*hypomorphs, or a *dun1*Δ can be reduced by as much as 46% of a control (Zhao et al., 2001; Fasullo et al., 2010; Hoch et al., 2013). Notably, all of these mutants are viable, which indicates that that genome duplication is possible even when dNTP levels fall below those that we observed in *mec1-4* cells. We also found that *mec1-4* cells in a SK1 background had ∼30% higher dNTP levels compared to a *MEC1* strain in a different genetic background of a comparably sized genome (∼17 Mb), which was used as a control in the above mentioned *mec1-/rad53-* hypomorph studies (Fig. 4B; 390 pmoles vs 296 pmoles/10^8^ cells). Taken together these observations strongly suggest that the dNTP pool in a *mec1-4* strain would have been sufficient to support genome duplication under normal condition, and therefore was unlikely to be the sole cause of the arrest. The ratio among four different dNTPs in *mec1-4* was comparable to *MEC1* (Fig. 4C), ruling out the possibility that the replication block is due to imbalance in dNTP precursors (Kumar et al., 2010).

To the best of our knowledge, these are the first direct dNTP pool measurements in a strain expressing a lethal *mec1* allele without altering Sml1 or RNR activity. While the results confirm a Mec1's role in promoting dNTP synthesis, they reveal that loss of this function is unlikely to be a direct cause of the *mec1* replication arrest. To date, ∼80 direct targets of Mec1 have been identified. They include components of the RPA complex (Rfa1 and Rfa2; Smolka et al., 2007), the GINS complex (Psf1; De Piccoli et al., 2012), and the MCM-helicase complex (Mcm4 and Mcm6; Randell et al., 2010), all of which are directly involved in DNA replication. Therefore, inactivation of Mec1 might result in a system-wide failure in genome duplication stemming from inability to phosphorylate key components of the replication machinery. Under such condition, DNA replication might become acutely sensitive to dNTP levels, whereby even a modest reduction in dNTP pool, which would not impair normal genome duplication, triggers a permanent arrest.

Most known suppressors of *mec1* lethality are involved in dNTP synthesis or its regulation (e.g. Desany et al., 1998; Zhao et al., 1998; Tsaponina et al., 2011). In yeast, the rate of replication fork progression correlates with the dNTP pool size (Malinsky et al., 2001; Sabouri et al., 2008; Odsbu et al., 2009; Poli et al., 2012). Furthermore, elevated levels of dNTP can promote fork progression through DNA lesions that normally block its progression (Malinsky et al., 2001; Sabouri et al., 2008; Odsbu et al., 2009; Poli et al., 2012). These observations suggest that *GIS2* suppression of *mec1-4* acts also via its effects on dNTP levels. Gis2 regulates protein levels of several hundred target mRNAs that are enriched in ribosome assembly, chromatin structure, GTPase activity, and stress signalling (Sammons et al., 2011; Scherrer et al., 2011). However, none of the well-known components of the Mec1 signalling network (e.g. *SML1*, *DUN1*, *RNR1-4*, *HUG1*, *DIF1*, *RAD53*, *RAD9*, or *TEL1*) is targeted by Gis2. Therefore, the mechanism underlying the *GIS2-*dependent dNTP pool increase is likely to be pleiotropic and indirect.

Current observations are reminiscent of the effects of dNTPs on mammalian genome duplication, in particular, with regard to oncogene-induced replication stress: It was shown that many consequences of the stress (e.g. DNA damage, fragile site expression, genome instability, and/or cell death) can be either exacerbated or rescued by changes in dNTP levels (Aird et al., 2013; Mannava et al., 2013; Olcina et al., 2013; Lopez-Contreras et al., 2015). Evidence presented above provides a fresh insight into the phenomenon that even a modest change in dNTP availability could result in a profound consequence.

## MATERIALS AND METHODS

### Yeast strains and media

All strains were of the SK1 background unless noted (Table S1). Hydroxylamine mutagenesis of *MEC1* and isolation of the temperature sensitive *mec1-4* allele were previously described (Cha and Kleckner, 2002). Multi-copy suppressor screen for *mec1-4* is described in Fig. S1. To obtain a synchronous culture for cell cycle analysis, cultures grown to mid-log phase at 23°C (permissive temperature for *mec1-4*) were arrested with 5 µg/ml α-factor for 3 h before being released to fresh YPD [1% (w/v) yeast extract, 2% (w/v) bacto-peptone, 2% (w/v) glucose] media at the indicated temperature.

### Fluorescence activated cell scan (FACS) analysis

Cells were fixed [40% (v/v) ethanol, 0.1 M sorbitol] for 3 h and incubated overnight at 37°C with RNase solution (50 mM Tris-HCl pH 7.5, 100 µg/ml RNaseA). The next day, the cells were treated with 500 µl of pepsin solution (50 mM HCl, 5 mg/ml pepsin) for 5 min or longer at room temperature being resuspended in 1 ml SYTOX solution (50 mM Tris-HCl pH 7.5, 1 µM SYTOX Green; Invitrogen, Molecular Probe). After an overnight incubation at 4°C, samples were analysed on a Becton Dickinson Flow Cytometer (Hashash et al., 2012).

### Western blotting and antibodies

Whole cell extracts from ∼10^7^–10^8^ cells were prepared from cell suspensions in 20% tricholoroacetic acid (TCA) by agitation with glass beads. Precipitated proteins were solubilized in SDS-PAGE sample butter and analysed by SDS-PAGE and western blotting. Antibodies for western blotting were mouse monoclonal α-HA (1:1000; NIMR, London, UK), α-MYC (1:1000; NIMR, London, UK), and rat monoclonal α-tubulin (1:5000; Abcam).

### Measurement of dNTP levels

NTP and dNTP extraction and quantification were performed as described (Jia et al., 2015).

## References

[BIO015347C1] Abraham R. (2001). Cell cycle checkpoint signaling through the ATM and ATR kinases. *Genes Dev.* 15, 2177-2196. 10.1101/gad.914401 11544175

[BIO015347C2] Aird K. M., Zhang G., Li H., Tu Z., Bitler B. G., Garipov A., Wu H., Wei Z., Wagner S. N., Herlyn M. et al. (2013). Suppression of nucleotide metabolism underlies the establishment and maintenance of oncogene-induced senescence. *Cell Rep.* 3, 1252-1265. 10.1016/j.celrep.2013.03.004 23562156 PMC3840499

[BIO015347C3] Allen J. B., Zhou Z., Siede W., Friedberg E. C. and Elledge S. J. (1994). The SAD1/RAD53 protein kinase controls multiple checkpoints and DNA damage-induced transcription in yeast. *Genes Dev.* 8, 2401-2415. 10.1101/gad.8.20.2401 7958905

[BIO015347C4] Balciunas D. and Ronne H. (1999). Yeast genes GIS1-4: multicopy suppressors of the Gal^−^ phenotype of snf1 wrb8/10/11 cells. *Mol. Gen. Genet.* 262, 589-599. 10.1007/s004380051121 10628841

[BIO015347C5] Brown E. and Baltimore D. (2000). ATR disruption leads to chromosomal fragmentation and early embryonic lethality. *Genes Dev.* 14, 397-402. 10691732 PMC316378

[BIO015347C6] Carballo J. A., Johnson A. L., Sedgwick S. G. and Cha R. S. (2008). Phosphorylation of the axial element protein Hop1 by Mec1/Tel1 ensures meiotic interhomolog recombination. *Cell* 132, 758-770. 10.1016/j.cell.2008.01.035 18329363

[BIO015347C7] Casper A. M., Nghiem P., Arlt M. F. and Glover T. W. (2002). ATR regulates fragile site stability. *Cell* 111, 779-789. 10.1016/S0092-8674(02)01113-3 12526805

[BIO015347C8] Cha R. S. and Kleckner N. (2002). ATR homolog Mec1 promotes fork progression, thus averting breaks in replication slow zones. *Science* 297, 602-606. 10.1126/science.1071398 12142538

[BIO015347C9] Chabes A., Georgieva B., Domkin V., Zhao X., Rothstein R. and Thelander L. (2003). Survival of DNA damage in yeast directly depends on increased dNTP levels allowed by relaxed feedback inhibition of ribonucleotide reductase. *Cell* 112, 391-401. 10.1016/S0092-8674(03)00075-8 12581528

[BIO015347C10] De Piccoli G., Katou Y., Itoh T., Nakato R., Shirahige K. and Labib K. (2012). Replisome stability at defective DNA replication forks is independent of S phase checkpoint kinases. *Mol. Cell* 45, 696-704. 10.1016/j.molcel.2012.01.007 22325992

[BIO015347C11] Desany B. A., Alcasabas A. A., Bachant J. B. and Elledge S. J. (1998). Recovery from DNA replicational stress is the essential function of the S-phase checkpoint pathway. *Genes Dev.* 12, 2956-2970. 10.1101/gad.12.18.2956 9744871 PMC317167

[BIO015347C12] Eykelenboom J. K., Harte E. C., Canavan L., Pastor-Peidro A., Calvo-Asensio I., Lorens-Agost M. and Lowndes N. F. (2013). ATR activates the S-M checkpoint during unperturbed growth to ensure sufficient replication prior to mitotic onset. *Cell Rep.* 5, 1095-1107. 10.1016/j.celrep.2013.10.027 24268773

[BIO015347C13] Fasullo M., Tsaponina O., Sun M. and Chabes A. (2010). Elevated dNTP levels suppress hyper-recombination in *Saccharomyces cerevisiae* S-phase checkpoint mutants. *Nucleic Acids Res.* 38, 1195-1203. 10.1093/nar/gkp1064 19965764 PMC2831302

[BIO015347C14] Hashash N., Johnson A. L. and Cha R. S. (2012). Topoisomerase II- and condensin-dependent breakage of MEC1ATR-sensitive fragile sites occurs independently of spindle tension, anaphase, or cytokinesis. *PLoS Genet.* 8, e1002978 10.1371/journal.pgen.1002978 23133392 PMC3486896

[BIO015347C15] Hoch N. C., Chen E. S.-W., Buckland R., Wang S.-C., Fazio A., Hammet A., Pellicioli A., Chabes A., Tsai M.-D. and Heierhorst J. (2013). Molecular basis of the essential s phase function of the rad53 checkpoint kinase. *Mol. Cell. Biol.* 33, 3202-3213. 10.1128/MCB.00474-13 23754745 PMC3753913

[BIO015347C16] Jia S., Marjavaara L., Buckland R., Sharma S. and Chabes A. (2015). Determination of deoxyribonucleoside triphosphate concentrations in yeast cells by strong anion-exchange high-performance liquid chromatography coupled with ultraviolet detection. *Methods Mol. Biol.* 1300, 113-121. 10.1007/978-1-4939-2596-4_8 25916709

[BIO015347C17] Kato R. and Ogawa H. (1994). An essential gene, *ESR1*, is required for mitotic cell growth, DNA repair and meiotic recombination in Saccharomyces cerevisiae. *Nucleic Acids Res.* 22, 3104-3112. 10.1093/nar/22.15.3104 8065923 PMC310282

[BIO015347C18] Kumar D., Viberg J., Nilsson A. K. and Chabes A. (2010). Highly mutagenic and severely imbalanced dNTP pools can escape detection by the S-phase checkpoint. *Nucleic Acids Res.* 38, 3975-3983. 10.1093/nar/gkq128 20215435 PMC2896522

[BIO015347C19] Lee Y. D., Wang J., Stubbe J. and Elledge S. J. (2008). Dif1 is a DNA-damage-regulated facilitator of nuclear import for ribonucleotide reductase. *Mol. Cell* 32, 70-80. 10.1016/j.molcel.2008.08.018 18851834 PMC3245869

[BIO015347C20] Liquori C. L., Ricker K., Moseley M. L., Jacobsen J. F., Kress W., Naylor S. L., Day J. W. and Ranum L. P. W. (2001). Myotonic dystrophy type 2 caused by a CCTG expansion in intron 1 of ZNF9. *Science* 293, 864-867. 10.1126/science.1062125 11486088

[BIO015347C21] Lopez-Contreras A. J., Specks J., Barlow J. H., Ambrogio C., Desler C., Vikingsson S., Rodrigo-Perez S., Green H., Rasmussen L. J., Murga M. et al. (2015). Increased Rrm2 gene dosage reduces fragile site breakage and prolongs survival of ATR mutant mice. *Genes Dev.* 29, 690-695. 10.1101/gad.256958.114 25838540 PMC4387711

[BIO015347C22] Malinsky J., Koberna K., Stanek D., Masata M., Votruba I. and Raska I. (2001). The supply of exogenous deoxyribonucleotides accelerates the speed of the replication fork in early S-phase. *J. Cell Sci.* 114, 747-750. 11171380 10.1242/jcs.114.4.747

[BIO015347C23] Mannava S., Moparthy K. C., Wheeler L. J., Natarajan V., Zucker S. N., Fink E. E., Im M., Flanagan S., Burhans W. C., Zeitouni N. C. et al. (2013). Depletion of deoxyribonucleotide pools is an endogenous source of DNA damage in cells undergoing oncogene-induced senescence. *Am. J. Pathol.* 182, 142-151. 10.1016/j.ajpath.2012.09.011 23245831 PMC3532713

[BIO015347C24] Matsuoka S., Huang M. and Elledge S. J. (1998). Linkage of ATM to cell cycle regulation by the Chk2 protein kinase. *Science* 282, 1893-1897. 10.1126/science.282.5395.1893 9836640

[BIO015347C25] Odsbu I., Morigen and Skarstad K. (2009). A reduction in ribonucleotide reductase activity slows down the chromosome replication fork but does not change its localization. *PLoS ONE* 4, e7617 10.1371/journal.pone.0007617 19898675 PMC2773459

[BIO015347C26] Olcina M. M., Foskolou I. P., Anbalagan S., Senra J. M., Pires I. M., Jiang Y., Ryan A. J. and Hammond E. M. (2013). Replication stress and chromatin context link ATM activation to a role in DNA replication. *Mol. Cell* 52, 758-766. 10.1016/j.molcel.2013.10.019 24268576 PMC3898930

[BIO015347C27] Perry J. and Kleckner N. (2003). The ATRs, ATMs, and TORs are giant HEAT repeat proteins. *Cell* 112, 151-155. 10.1016/S0092-8674(03)00033-3 12553904

[BIO015347C28] Poli J., Tsaponina O., Crabbé L., Keszthelyi A., Pantesco V., Chabes A., Lengronne A. and Pasero P. (2012). dNTP pools determine fork progression and origin usage under replication stress. *EMBO J.* 31, 883-894. 10.1038/emboj.2011.470 22234185 PMC3280562

[BIO015347C29] Rajavashisth T., Taylor A., Andalibi A., Svenson K. and Lusis A. (1989). Identification of a zinc finger protein that binds to the sterol regulatory element. *Science* 245, 640-643. 10.1126/science.2562787 2562787

[BIO015347C30] Randell J. C. W., Fan A., Chan C., Francis L. I., Heller R. C., Galani K. and Bell S. P. (2010). Mec1 is one of multiple kinases that prime the Mcm2-7 helicase for phosphorylation by Cdc7. *Mol. Cell* 40, 353-363. 10.1016/j.molcel.2010.10.017 21070963 PMC3021128

[BIO015347C31] Sabouri N., Viberg J., Goyal D. K., Johansson E. and Chabes A. (2008). Evidence for lesion bypass by yeast replicative DNA polymerases during DNA damage. *Nucleic Acids Res.* 36, 5660-5667. 10.1093/nar/gkn555 18772226 PMC2553575

[BIO015347C32] Sammons M. A., Samir P. and Link A. J. (2011). Saccharomyces cerevisiae Gis2 interacts with the translation machinery and is orthogonal to myotonic dystrophy type 2 protein ZNF9. *Biochem. Biophys. Res. Commun.* 406, 13-19. 10.1016/j.bbrc.2011.01.086 21277287

[BIO015347C33] Scherrer T., Femmer C., Schiess R., Aebersold R. and Gerber A. P. (2011). Defining potentially conserved RNA regulons of homologous zinc-finger RNA-binding proteins. *Genome Biol.* 12, R3 10.1186/gb-2011-12-1-r3 21232131 PMC3091301

[BIO015347C34] Smolka M. B., Albuquerque C. P., Chen S. H. and Zhou H. (2007). Proteome-wide identification of in vivo targets of DNA damage checkpoint kinases. *Proc. Natl. Acad. Sci. USA* 104, 10364-10369. 10.1073/pnas.0701622104 17563356 PMC1965519

[BIO015347C35] Tsaponina O., Barsoum E., Åström S. U. and Chabes A. (2011). Ixr1 is required for the expression of the ribonucleotide reductase Rnr1 and maintenance of dNTP pools. *PLoS Genet.* 7, e1002061 10.1371/journal.pgen.1002061 21573136 PMC3088718

[BIO015347C36] Wang Y., Xu H. P., Riggs M., Rodgers L. and Wigler M. (1991). byr2, a Schizosaccharomyces pombe gene encoding a protein kinase capable of partial suppression of the ras1 mutant phenotype. *Mol. Cell. Biol.* 11, 3554-3563. 10.1128/MCB.11.7.3554 2046669 PMC361098

[BIO015347C37] Weinert T. A., Kiser G. L. and Hartwell L. H. (1994). Mitotic checkpoint genes in budding yeast and the dependence of mitosis on DNA replication and repair. *Genes Dev.* 8, 652-665. 10.1101/gad.8.6.652 7926756

[BIO015347C38] Yao R., Zhang Z., An X., Bucci B., Perlstein D. L., Stubbe J. and Huang M. (2003). Subcellular localization of yeast ribonucleotide reductase regulated by the DNA replication and damage checkpoint pathways. *Proc. Natl. Acad. Sci. USA* 100, 6628-6633. 10.1073/pnas.1131932100 12732713 PMC164498

[BIO015347C39] Zhao X. and Rothstein R. (2002). The Dun1 checkpoint kinase phosphorylates and regulates the ribonucleotide reductase inhibitor Sml1. *Proc. Natl. Acad. Sci. USA* 99, 3746-3751. 10.1073/pnas.062502299 11904430 PMC122595

[BIO015347C40] Zhao X., Muller E. G. D. and Rothstein R. (1998). A suppressor of two essential checkpoint genes identifies a novel protein that negatively affects dNTP pools. *Mol. Cell* 2, 329-340. 10.1016/S1097-2765(00)80277-4 9774971

[BIO015347C41] Zhao X., Chabes A., Domkin V., Thelander L. and Rothstein R. (2001). The ribonucleotide reductase inhibitor Sml1 is a new target of the Mec1/Rad53 kinase cascade during growth and in response to DNA damage. *EMBO J.* 20, 3544-3553. 10.1093/emboj/20.13.3544 11432841 PMC125510

